# Gynecological and Obstetrical Society of Baltimore

**Published:** 1894-01

**Authors:** William S. Gardner

**Affiliations:** 513 Park avenue


### GYNECOLOGICAL AND OBSTETRICAL SOCIETY OF BALTIMORE.

### OCTOBER MEETING.

The President, Dr. T. A. Ashby, in the chair.

DR. J. MASON HUNDLEY read a paper upon the "Treatment of Incomplete Abortions," of which the following is an abstract:

He uses the finger in all cases of incomplete abortions to remove adherent secundings, etc. Where after exploration with the finger, there is only found small portions of tissue, he sees no objection to the use of the curette. But when called to a case of incomplete abortion, he has universally found that the finger can be used with ease and efficiency where there is an appreciable amount of tissue retained within the uterus. The practice of blindly curetting a puerperal uterus without knowing the condition within its cavity, cannot, in his judgment, be too strongly condemned. There is a condition of the uterus mucous membrane following incomplete abortions, where the only symptom present is hemorrhage, with the uterus about its normal size or but slightly enlarged. If the uterus be curetted there will be gotten away many minute particles of tissue. This class of cases do not come strictly under the head of incomplete abortions.

He cited several cases where operators had attempted to curette uteri containing adherent placental tissue—the hemorrhage continuing, another operator was called in, and when the finger was introduced in the uterine cavity it was found filled with fœtal remains. These cases with many others, proves to his mind that it is impossible to appreciate the amount of retained tissue in a puerperal uterus at the end of a curette. He further believes that the traumatism induced by the curette in these cases, adds greatly to the danger of septic infection which can easily be made clear. Suppose after a curettement there remain pieces of tissue, as is often done, these pieces will undergo decomposition. With a denuded surface, which we have where we scrape blindly about in a puerperal uterus, absorption can and certainly does occur more readily.

Then another danger is the liability of penetrating the uterine wall. And it is done in the non-puerperal uterus, it seems to him very much easier of accomplishment in one that is soft and yielding as is found in these cases.

DR. THOMAS A. ASHBY: I think this is a very important subject. I have had three cases in the last three weeks. In the last eighteen months or two years I have had, I think, from twenty-five to thirty cases in consultation, and in my own work. I have used the finger to remove what was left behind when it was possible to do so, but in many cases it is impossible, and in these cases I have used the curette, and have not found the curette dangerous and *have* found it very efficient. I think there is some danger when great force is used, but I have had no trouble, and can recall no case where the patient has had pus tubes or other septic troubles as the result. I have used the curette on septic cases, and the temperature fell at once. I have met with no patients injured by other operators. My method is first to wash out the vagina with a bichloride solution, then curette, and wash out the uterus cavity with a $\frac{1}{5000}$ or a $\frac{1}{1000}$ bichloride solution. All of my cases have recovered.

DR. J. EDWIN MICHAEL: It is difficult for me to fix my position when two extremes are presented as they have been here to-night. I think I can take a middle ground.

The small curette is not only dangerous but inefficient. The large curette is not dangerous and is very efficient. I use it frequently, and I think I can tell the difference between the uterine wall and the placental tisues with the curette, and I also think you can clean the uterine wall more thoroughly with the curette than with the finger. If the staff of the curette is of some malleable metal, as it should be, you can so change the position so as to reach all parts of the uterine wall. It is sometimes impossible to get the finger all over the uterine wall.

DR. THOMAS OPHIE: I do not remember to have had a bad result from the use of the curette in abortions. It is unnecessary to cut away any of the wall of the uterus with a sharp curette in early abortions, since as yet the attachments of the ovum are not strong, and the rule is, it comes off in tact. Thomas' dull wire curette is most suitable in such cases. Ergot does more harm than good. It promotes tonic contraction of the cervical sphincters, and useless delay is occasioned in awaiting its action. All delay is wrong. Abortions when inevitable should be regarded as demanding immediate operative removal, since the two dangers, hemorrhage and sepsis, confront us. The finger is safest but does not succeed in all cases ; the same may be said for the curette. When both of these methods fail, we can resort to the parallel-bar dilator. The cervix having been properly dilated, the curette can be successfully resorted to in nearly, if not all cases. These dilators are required later in pregnancy, after the placenta is more fully formed and more difficult of detachment. Here the sharp curette is often best for the removal of the strongly adherent parts of the placenta.

DR. WILLIAM P. CHUNN: I did not hear Dr. Hundley's paper, but from the discussion I infer that in some cases he is opposed to the use of the curette. I have used that instrument frequently, and so far without objectional results. The curette, to be effective, should be adapted to the size of the cavity of the uterus. In treating a case of incomplete abortion where dilitation is needed, the question arises, "What is the best way of opening up the cervix?" Where the cervix is soft, a meehanical dilator may be best. If the cervix is hard, a tent would soften and dilate better. I prefer to use a tent as large as possible as a single tent is easier to clean and introduce than a number of smaller ones.

WILLIAM S. GARDNER,
513 Park avenue. Secretary.

